# Repeatability and Cross-Domain Agreement of Pyramidal-Sensor Ocular Wavefront Measurements in Myopic Eyes: A Registry Validation of OSIRIS

**DOI:** 10.1167/tvst.15.10.49

**Published:** 2026-10-29

**Authors:** Ivan Gabrić, Ivan Samuel Modrić, Filip Grulović

**Affiliations:** 1 Eye Clinic Svjetlost, Zagreb, Croatia; 2 University of Zagreb School of Medicine, Zagreb, Croatia

**Keywords:** pyramidal wavefront sensor, aberrometry, repeatability, higher-order aberrations (HOAs), spherical aberration, agreement, quality control (QC)

## Abstract

**Purpose:**

The purpose of this study was to quantify within-session repeatability of ocular wavefront metrics from a pyramidal wavefront-sensor aberrometer (OSIRIS; CSO), to define a pupil-support and zero-code gate for 6-mm analysis, and to assess agreement between OSIRIS whole-eye and MS-39 total-corneal wavefront.

**Methods:**

Retrospective single-center registry analysis of myopic and myopic astigmatic eyes. Within-subject standard deviation (SD), repeatability coefficient (RC = 2.77 × SD), and intraclass correlation coefficient ICC (1.1) were computed across within-session scan clusters with cluster-bootstrap confidence intervals (CIs). Agreement used Bland-Altman limits and Lin concordance correlation coefficient (CCC); and astigmatism used Thibos power vectors.

**Results:**

Across 10,416 sessions from 2533 eyes, single-scan repeatability was excellent: 4-mm higher-order aberration (HOA) root mean square (RMS), ICC = 0.987 (RC = 0.033 µm); pupil-supported 6-mm HOA RMS ICC = 0.992 (RC = 0.050 µm); and 6-mm spherical aberration ICC = 0.997. Preoperative eyes were the noisier stratum (6-mm HOA RC = 0.065 vs. 0.039 postoperatively). Diameters below 6 mm gave entirely zero-coded 6-mm exports in 96.8% of sessions. Omitting scan-selection worsened 6-mm HOA RC to 0.257 µm. Whole-eye and total-corneal values correlated but were not interchangeable (6-mm HOA RMS CCC = 0.623). Objective and manifest refraction were consistent (4-mm sphere CCC = 0.965), a rounding-limited consistency check.

**Conclusions:**

In myopic eyes, OSIRIS shows excellent within-session repeatability under the described acquisition and quality-control (QC) workflow. Six-millimeter analysis requires pupil-diameter and zero-code gating. Whole-eye and total-corneal wavefront are not interchangeable.

**Translational Relevance:**

Within-session precision measured in routine high-volume practice, with a pupil-diameter and zero-code quality rule, defines the smallest same-session change distinguishable from measurement variability, informing wavefront-guided planning, lens selection, and post-refractive follow-up.

## Introduction

Ocular wavefront aberrometry underlies several high-value decisions in refractive and cataract surgery: wavefront-guided and wavefront-optimized ablation planning, selection of aspheric and presbyopia-correcting intraocular lenses, toric alignment, and the assessment of visual complaints after corneal refractive surgery. These decisions frequently hinge on differences of a tenth of a micrometer or less in higher-order aberration (HOA) magnitude. The measurement noise of the aberrometer is therefore itself a clinical parameter. A measured change is meaningful only when it exceeds the repeatability coefficient of the instrument. This principle has an explicit surgical corollary: several authors have argued that an eye should be considered a candidate forwavefront-guided correction of a given aberration only when that aberration exceeds the device repeatability limit, because a value below the limit cannot be distinguished from measurement noise, and treating noise risks adding rather than removing the aberration.^1^

OSIRIS uses a 4-faced pyramidal wavefront sensor, an approach analogous to the Foucault knife-edge test, that analyzes light intensity across 4 sub-aperture images of the entrance pupil and reconstructs the wavefront at up to approximately 45,000 sampling points at the maximum pupil. This is a distinct acquisition principle from the Hartmann-Shack lenslet arrays used by most commercial aberrometers (for example, iDesign and WASCA), from the automated retinoscopy or slit-skiascopy principle of the OPD-Scan III, from the ray-tracing principle of the iTrace, and from the Scheimpflug-plus-Hartmann-Shack hybrid of the Pentacam AXL Wave. The high spatial sampling of the pyramidal sensor is intended to improve reconstruction of fine higher-order structure. Bench and small-sample clinical validations of pyramidal-sensor aberrometry have reported low measurement error and high repeatability.^2^^–^^4^

Two gaps remain. First, every published aberrometer-repeatability study, across all technologies, rests on tens of eyes and a small number of consecutive scans acquired in a controlled setting (Table 1). None reports the repeatability that a device actually achieves across a live, high-volume clinical registry, where acquisition quality, pupil size, fixation, and device-exported data completeness vary far more than in a scripted precision study. Second, large-pupil (6-mm) wavefront analysis depends on the pupil being large enough to support the requested aperture, and devices commonly export a zero-coded or null wavefront when it is not. Whether such exports are silently propagated into analysis is rarely examined, yet it directly determines the validity of any 6-mm result.

**Table 1.** Prior Aberrometer Repeatability and Agreement Literature (Comparator Data)

Study	Ref	Device	Cohort / Design	Key Repeatability	Notes
Pyramidal Wavefront Sensor (Same Technology as OSIRIS)					
Singh et al. 2019	2	Osiris	Model eye + 1 presbyopic eye, 10 repeats	SA RMS error 0.01 µm, coma RMS error 0.02 µm (model eye); sphere test-retest SD 0.026 D (model), 0.039 D (eye)	Bench validation
Plaza-Puche/Salerno et al. 2019	3	Osiris	53 normal eyes, 3 scans	ICC ≥0.798 at 4 mm (Z(4,4) 0.683); ICC ≥0.850 at 6 mm	First clinical repeatability study
Ibrahim/Assaf/Awwad et al. 2024	4	Osiris (pyramidal)	76 normal + 72 keratoconus eyes, 3 scans	ICC total LOA and HOA >0.9 both groups; keratoconus (Kmax <50 D) Sw: HOA 0.062 µm, horizontal coma 0.029 µm, vertical coma 0.056 µm, SA 0.022 µm	Most directly comparable clinical benchmark
Yang et al. 2024	11	Osiris-T (pyramidal) vs OPD-Scan III	70 healthy eyes, 3 scans, 4 and 6 mm	High agreement for ocular, corneal and internal aberrations; 95% LoA up to 1.06 µm (4 mm) and 1.13 µm (6 mm); authors concluded the two devices may be considered interchangeable	Same-domain (ocular vs ocular) device comparison, unlike the cross-domain comparison here
This study	–	OSIRIS	2,533 eyes; ∼10,400 sessions	4-mm HOA RC 0.033 µm (ICC 0.987); 6-mm HOA RC 0.050 µm (ICC 0.992); 6-mm SA RC 0.028 µm (ICC 0.997)	Registry scale; QC gate
Study	Ref	Device (Principle)	Cohort / Design	Key Repeatability	Agreement

Hartmann-Shack, Ray-Tracing, Slit-Skiascopy, and Scheimpflug-Hybrid Devices					
Xu/Wu et al. 2018	9	iTrace (ray tracing)	92 normal eyes, 3 scans, 4 mm	Whole-eye HOA repeatability limit ≤0.105 µm, ICC ≥0.805; whole-eye SA Sw 0.016–0.019 µm, ICC 0.992–0.995; whole-eye coma ICC 0.989–0.992	iTrace vs KR-1 W whole-eye HOA LoA 0.46 µm, SA 0.19 µm: not interchangeable
Xu/Wu et al. 2018	9	Topcon KR-1 W (Hartmann-Shack)	92 normal eyes, 3 scans, 4 mm	Whole-eye HOA repeatability limit ≤0.079 µm, ICC ≥0.761; whole-eye tHOA ICC ≥0.957; SA Sw 0.027–0.031 µm; coma ICC 0.931–0.945	Reproducibility lower than repeatability (many ICC <0.75 except SA)
Wan et al. 2022	10	Pentacam AXL Wave (Scheimpflug + HS); iTrace; OPD-Scan III	47 eyes, 3 scans, 4 mm	Whole-eye HOA RMS repeatability similar across all three; Pentacam AXL Wave and OPD-Scan III better than iTrace for total RMS (P ≤0.02); SA comparable	Most individual Zernike terms not interchangeable across devices
Lopez-Miguel et al. 2013	12	Topcon KR-1 W (HS)	Normal eyes, 6 mm	Whole-eye tHOA ICC 0.902; SA ICC 0.902 (repeatability), 0.793 (intersession)	–
Pinero et al. 2011	13	Integrated aberrometer	Normal eyes, 4 and 6 mm	Whole-eye tHOA ICC 0.795 (4 mm), 0.864 (6 mm); SA ICC 0.949; coma ICC 0.673	–
Visser et al. 2011	14	4 aberrometers (Zywave, iRx, Keratron, iTrace)	Normal eyes	Comparability and repeatability vary by device; iTrace SA 0.064 ± 0.076 µm	Devices not interchangeable
Rozema et al. 2006	15	6 aberrometers incl. WASCA (HS)	44 healthy eyes	WASCA showed consistently low variance (high repeatability); ray-tracing devices reported higher HOA magnitudes	Significant inter-device differences
WASCA vs OPD-Scan comparison	16	WASCA (Zeiss, Hartmann-Shack)	Normal eyes	WASCA coefficient of repeatability 0.008 to 0.022 µm across Zernike coefficients	WASCA Zernike distributions normal for all terms; OPD-Scan only for selected terms
Lopez-Miguel et al. 2012	1	Hartmann-Shack (Zywave)	Normal eyes	Whole-eye SA Sw 0.186 µm; proposed that only eyes with HOA above the repeatability limit are wavefront-guided candidates	Precision limits of wavefront correction
iDesign / iDesign 2.0 (high-resolution Hartmann-Shack)	–	AMO / Johnson & Johnson Vision	–	No dedicated within-subject repeatability study (Sw, RC, or ICC) identified; published iDesign reports are surgical-outcome or WaveScan-comparison studies. Qualitatively, iDesign gives more myopic values and lower total, third-, fourth-, and fifth-order HOA than WaveScan	Honest evidence gap: iDesign precision is not directly benchmarkable from published data

Values are as reported in each source. Repeatability metrics differ across studies (Sw, repeatability limit 2.77 x Sw, or ICC). HOA metrics at 4–6 mm are prioritized. This table supports the Discussion; it is not a head-to-head comparison, since designs and cohorts differ.

Among the metrics studied here, the Micro Aberration Scatter Index (MASI) deserves a specific rationale: this device-derived composite was recently shown to be associated with contrast sensitivity after keratorefractive surgery,^5^ making it a candidate objective quality-of-vision proxy, and its measurement precision is a prerequisite for any longitudinal use.

This study addresses both gaps in a single registry. The aims were: first, to quantify the within-session repeatability of OSIRIS whole-eye wavefront metrics at 4-mm and 6-mm apertures across thousands of sessions; second, to define and validate a pupil-support and zero-code quality gate for 6-mm analysis; and third, to quantify agreement between OSIRIS whole-eye wavefront and MS-39 total-corneal wavefront, and between OSIRIS objective and clinical manifest refraction. The third aim is not an instrument comparison for its own sake: wavefront-guided treatment strategies are built on whole-eye measurements whereas corneal- and topography-guided strategies are built on corneal data, so whether the two domains can substitute for one another in planning is a clinically consequential question.

## Methods

### Study Design and Setting

This was a retrospective analysis of a prospectively maintained single-center refractive surgery registry at Eye Clinic Svjetlost, Zagreb, Croatia. The study adhered to the Declaration of Helsinki and was approved by the institutional ethics committee (approval no. KS-OSIRIS-Reg01, approved June 25, 2026). The registry was anonymized for analysis, with separate consents for the procedure and for retrospective use of de-identified registry data.

### Devices and Acquisition

Whole-eye wavefront aberrometry was performed with the OSIRIS pyramidal wavefront-sensor aberrometer (CSO, Florence, Italy), operated under Phoenix software version 4.1.4.7. The device uses a near-infrared light-emitting-diode source (approximately 850 nm) and a 4-faced pyramidal sensor sampling the wavefront at up to approximately 45,000 points at the maximum pupil. Total-corneal wavefront, used for the agreement analysis, was derived from the MS-39 anterior-segment optical coherence tomographer with integrated Placido topography (CSO), operated under the same Phoenix software version. All captures for both devices were acquired under this version. All acquisitions followed a single standardized registry workflow: measurements were taken in a darkened room with no additional illumination and without pharmacologic dilation, and aberrometry was always performed first in the visit, before topography, tomography, and pupillometry. Each eye was scanned a minimum of three times, and the sequence was repeated if three acquisitions of acceptable quality were not obtained. All scans across the registry were performed by four highly trained optometrists, with repeated scans of a given eye within a session acquired by the same operator. Full protocol details are in Supplementary S1.

### Cohort and Eligibility

Eligible eyes were myopic and myopic astigmatic eyes with OSIRIS acquisitions between April 2022 and March 2026 undergoing keratorefractive lenticule extraction. Hyperopic eyes, eyes with previous corneal refractive surgery, and pseudophakic eyes were excluded; these populations are the subject of planned separate reports. The registry includes preoperative and postoperative visits (postoperative months 1, 3, 6, and 12), so the cohort spans untreated myopic eyes and the same eyes after lenticule extraction. This real-world span strengthens external validity for the myopic treatment pathway while limiting cohort homogeneity.

### Wavefront Metrics

Primary metrics were HOA RMS at 4-mm and 6-mm apertures, spherical aberration (Zernike Z(4.0); OSA index *j* = 12) at 6 mm, and objective spherical equivalent at 4 mm. Secondary metrics were coma RMS, trefoil RMS, the Strehl ratio, and the Micro Aberration Scatter Index (MASI),^5^ reported multiplied by 1000 following the registry convention. Zernike coefficients follow the OSA ordering and normalization standard.^6^ Analysis apertures are defined relative to the maximum wavefront capture diameter of each acquisition; the device automatically samples the largest available pupil up to a 9.0-mm ceiling. Aperture definitions and the full metric dictionary are in Supplementary S2 and S3.

### Session Definition and Scan Selection

A within-session repeatability cluster was defined as all repeat scans sharing patient, laterality, acquisition date, and acquisition time. Primary repeatability used the raw scans retained by the registry scan-selection workflow, which is the same set of scans the device and registry use to form clinical averages. Exact zero-coded wavefront exports were treated as missing rather than as true zeros and were excluded. Implausible values were removed by a robust rule (greater than 3.5 median absolute deviations from the within-session location for MASI); the number of excluded scans is reported.

### Pupil-Support and Zero-Code Gate

Pupil support at each aperture was defined from the maximum wavefront diameter of the scan. The prespecified 6-mm analysis gate required both a valid non-zero wavefront and a maximum wavefront diameter of at least 6.0 mm. The 4-mm gate required a valid non-zero wavefront. The value of the gate was then evaluated empirically in the Results section.

### Statistical Analysis

Within-subject standard deviation (SD) was estimated from an unbalanced one-way random-effects analysis of variance with patient-eye-session as the grouping factor. The repeatability coefficient was RC = 2.77 × SD, equivalent to 1.96 × sqrt(2) × SD, the interval within which 95% of the differences between 2 measurements of the same eye are expected to fall.^7^ The within-subject coefficient of variation was reported where the metric mean was well separated from zero. Single-scan reliability was quantified with ICC (1.1); the reliability of the mean of two and three scans was computed as ICC (1.2) and ICC (1.3), and the corresponding averaged-measurement repeatability coefficients were derived from the standard error of the mean. The one-way model assumes homoscedastic within-session variance across eyes. Because measurement variance could plausibly differ between untreated and operated eyes, all primary repeatability estimates were additionally stratified into preoperative and postoperative subsets, and the postoperative-to-preoperative SD ratio was estimated with bootstrap confidence intervals (CIs). The CIs for Sw, repeatability coefficient (RC), intraclass correlation coefficient (ICC), and Bland-Altman parameters were obtained by patient-level cluster bootstrap with 500 replicates, resampling patients to respect the clustered structure of the data.

Agreement between paired measurements was assessed with Bland-Altman mean bias and 95% limits of agreement (LoA),^8^ the Pearson correlation coefficient, the Spearman rank correlation coefficient, the Lin concordance correlation coefficient (CCC), and the absolute-agreement ICC. Proportional bias was tested by regressing the paired difference on the paired mean with patient-clustered Huber-White standard errors. Agreement of refraction between OSIRIS objective and clinical manifest values was assessed both on sphere and cylinder directly and, for astigmatism, on the Thibos power-vector components (J0, J45, and the spherical-equivalent M), which avoid the circular-scale problems of raw cylinder axis. Axis agreement was additionally reported as the acute angular difference between meridians and stratified by cylinder magnitude, because axis is ill-defined at low cylinder; clinical manifest axis was recorded only at the preoperative visit, so the astigmatism and axis agreement analyses are preoperative. A quality-control sensitivity analysis repeated all repeatability estimation using every numeric raw scan without registry selection and without zero-code exclusion, for direct comparison with the primary quality control (QC)-selected analysis. No imputation, sign correction, or left-eye mirroring was applied at any stage. Analyses were performed in Python software version 3.12 (pandas, numpy, scipy, statsmodels, and matplotlib).

## Results

### Cohort and Session Flow

The registry contained 2533 eyes and 30,915 raw OSIRIS scan rows. The scan-selection workflow retained 23,718 raw scans, forming 10,416 unique patient-laterality-date-time sessions, of which 9824 contained at least 2 selected scans (Fig. 1). The median number of scans per session was two. Preoperative refraction confirmed a uniformly myopic cohort: manifest spherical equivalent ranged from −12.75 to −0.60 diopter (D; mean = −3.83 ± 1.91 D, median = −3.50 D), sphere from −12.00 to +0.25 D (mean = −3.28 ± 1.96 D), and cylinder from −6.00 to 0.00 D (mean = −1.09 ± 1.03 D). No eye had a hyperopic spherical equivalent. The primary 4-mm HOA repeatability cohort comprised 9761 sessions (2377 eyes and 1225 patients). The pupil-supported 6-mm HOA cohort comprised 6020 sessions (1849 eyes and 999 patients).

**Figure 1.**
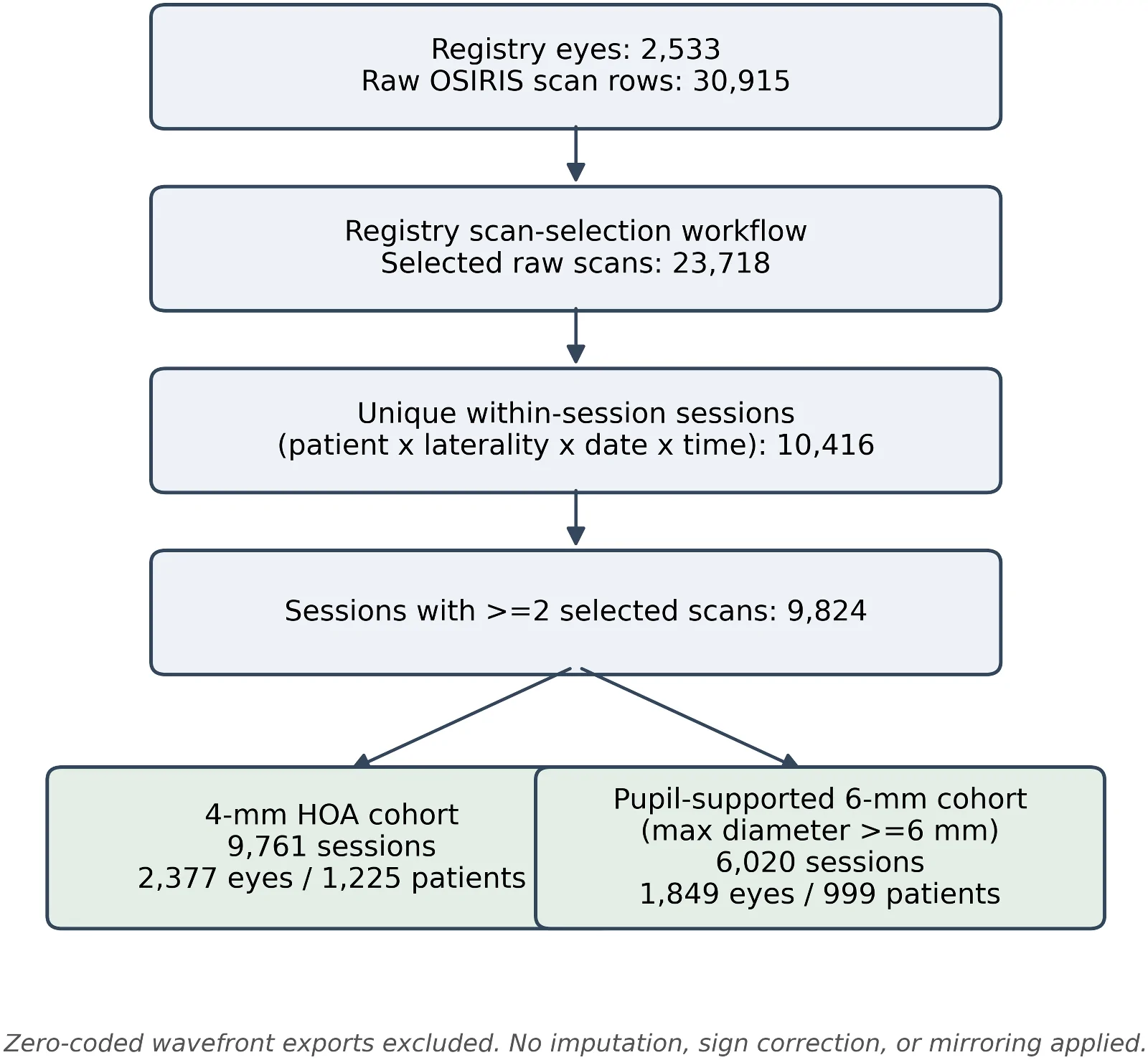
**Study flow.** Registry eyes through registry scan-selection to the within-session analysis cohorts.

### Primary Within-Session Repeatability

Within-session repeatability was excellent across all primary metrics (Table 2, Fig. 5). For 4-mm HOA RMS, SD was 0.012 µm, RC was 0.033 µm (95% CI = 0.030–0.036), and ICC (1.1) was 0.987 (95% CI = 0.983–0.990). For pupil-supported 6-mm HOA RMS, SD was 0.018 µm, RC was 0.050 µm (95% CI = 0.046–0.053), and ICC was 0.992 (95% CI = 0.990–0.994). Six-millimeter spherical aberration was the most repeatable HOA term, with SD = 0.010 µm, RC = 0.028 µm, and ICC = 0.997. Objective spherical equivalent at 4 mm had SD = 0.082 D, RC = 0.228 D, and ICC = 0.998. Coma and trefoil RMS at 4 mm had RC = 0.029 µm and 0.031 µm, respectively, with ICCs of 0.980 and 0.969. The composite MASI (×1000) had RC = 7.392 and ICC = 0.976. The Strehl ratio had ICC = 0.977 at 4 mm and 0.904 at 6 mm.

**Table 2.** Primary Within-Session Repeatability of OSIRIS Wavefront Metrics

Metric	Aperture	Sessions	Sw	RC (2.77 x Sw)	RC, Mean of 2	ICC(1,1)	95% CI (ICC)	CVw
HOA RMS	4 mm	9,761	0.012 µm	0.033 µm (0.030–0.036)	0.023 µm	0.987	0.983–0.990	6.29%
HOA RMS	6 mm (maxDiam ≥6)	6,020	0.018 µm	0.050 µm (0.046–0.053)	0.035 µm	0.992	0.990–0.994	3.98%
Spherical aberration	6 mm (maxDiam ≥6)	6,020	0.010 µm	0.028 µm (0.026–0.029)	0.020 µm	0.997	0.996–0.997	–
Coma RMS	4 mm	9,761	0.010 µm	0.029 µm	0.021 µm	0.980	0.974–0.984	11.08%
Trefoil RMS	4 mm	9,761	0.011 µm	0.031 µm	0.022 µm	0.969	0.962–0.975	13.18%
Objective SEQ	4 mm	9,761	0.082 D	0.228 D (0.155–0.318)	0.162 D	0.998	0.996–0.999	–
MASI (x1000)	central 3 mm	9,408	2.667	7.392 (5.033–9.116)	5.227	0.976	0.951–0.991	9.23%
Strehl ratio	4 mm	12,215	0.015	0.042	0.030	0.977	–	9.01%
Strehl ratio	6 mm	7,862	0.022	0.060	0.042	0.904	–	27.4%

CVw, within-subject coefficient of variation; ICC, intraclass correlation coefficient; RC, repeatability coefficient; Sw, within-subject standard deviation.

Full confidence intervals in the analysis package.

Stratified analysis did not support the assumption of a common within-session variance, and the direction of the difference was the opposite of a tear-film-driven expectation: preoperative eyes were the noisier stratum (Table 3). The postoperative-to-preoperative SD ratio was 0.68 (95% CI = 0.57–0.81) for 4-mm HOA RMS, 0.60 (95% CI = 0.52–0.67) for 6-mm HOA RMS, and 0.74 (95% CI = 0.66–0.83) for 6-mm spherical aberration, with no difference for objective spherical equivalent (1.05, 95% CI = 0.55–1.79). Pooled coefficients in Table 2 therefore sit between the stratum-specific values, and stratum-specific RCs from Table 3 should be used when judging change in a specific clinical context. The lower preoperative ICC despite larger SD also reflects the narrower between-eye spread of untreated myopic corneas; ICC is population-dependent, whereas SD and RC transfer across settings.

**Table 3.** Preoperative Versus Postoperative Within-Session Repeatability

Metric	Stratum	Sessions	Sw	RC (95% CI)	ICC(1,1)
HOA RMS, 4 mm (µm)	Preoperative	2,570	0.015	0.041 (0.037–0.046)	0.910
HOA RMS, 4 mm (µm)	Postoperative	7,191	0.010	0.028 (0.024–0.032)	0.993
HOA RMS, 6 mm (µm)	Preoperative	1,770	0.024	0.065 (0.059–0.072)	0.963
HOA RMS, 6 mm (µm)	Postoperative	4,250	0.014	0.039 (0.036–0.042)	0.996
Spherical aberration, 6 mm (µm)	Preoperative	1,770	0.012	0.033 (0.030–0.036)	0.989
Spherical aberration, 6 mm (µm)	Postoperative	4,250	0.009	0.024 (0.023–0.026)	0.998
Objective SEQ, 4 mm (D)	Preoperative	2,570	0.080	0.221 (0.178–0.265)	0.998
Objective SEQ, 4 mm (D)	Postoperative	7,191	0.084	0.231 (0.132–0.347)	0.987

Postoperative-to-preoperative Sw ratios: 0.68 (95% CI 0.57–0.81) for 4-mm HOA RMS, 0.60 (0.52–0.67) for 6-mm HOA RMS, 0.74 (0.66–0.83) for 6-mm spherical aberration, and 1.05 (0.55–1.79) for objective SEQ. Gating identical to Table 2; 6-mm strata are pupil-supported. CIs are patient-level cluster bootstrap.

### Gain From Averaging

Averaging 2 scans reduced the HOA RMS repeatability coefficient from 0.033 to 0.023 µm at 4 mm and from 0.050 to 0.035 µm at 6 mm. Averaging 3 scans reduced it further to 0.019 µm and 0.029 µm. The marginal gain from the third scan was small relative to the second, supporting a practical default of two scans per eye.

### Pupil-Support and Zero-Code Gate

The pupil-support gate was strongly justified by the data (Table 4). Among sessions with maximum wavefront diameter below 6 mm, only 3.2% yielded a valid 6-mm wavefront and 96.8% were entirely zero-coded at 6 mm. Among sessions with maximum wavefront diameter at or above 6 mm, 96.9% yielded a valid 6-mm wavefront and only 3.1% were entirely zero-coded (Fig. 2). A maximum wavefront diameter below 6 mm therefore almost guarantees an unusable 6-mm export. When 6-mm results were stratified by pupil support, the absolute within-session differences of the HOA metrics were not materially larger below 6 mm than at or above 6 mm (all *P* greater than 0.05 for HOA RMS, coma, and spherical aberration), whereas the lower-order spherical-equivalent difference was larger below 6 mm (*P* \< 0.001). This pattern supports gating on wavefront validity and availability rather than on any degradation of HOA repeatability, and it confirms that the gate protects against zero-coded and unsupported data rather than against a subtle loss of precision. The gate certifies availability and validity of the 6-mm export, not the trueness of the peripheral measurement. Within pupil-supported sessions, repeatability did not deteriorate toward the threshold: stratifying by the session minimum maximum-wavefront diameter, 6-mm HOA RC was 0.043 µm at 6.00 to 6.24 mm, 0.048 µm at 6.25 to 6.49 mm, 0.050 µm at 6.50 to 6.99 mm, and 0.052 µm at or above 7.00 mm. Sessions just above the gate were, if anything, the most repeatable.

**Table 4.** Pupil-Support Gate: 6-mm Wavefront Availability by Maximum Wavefront Diameter

Maximum Wavefront Diameter	Sessions	Valid 6-mm Wavefront	Entirely Zero-Coded at 6 mm
Below 6 mm	3,644	3.2%	96.8%
At or above 6 mm	6,764	96.9%	3.1%

**Figure 2.**
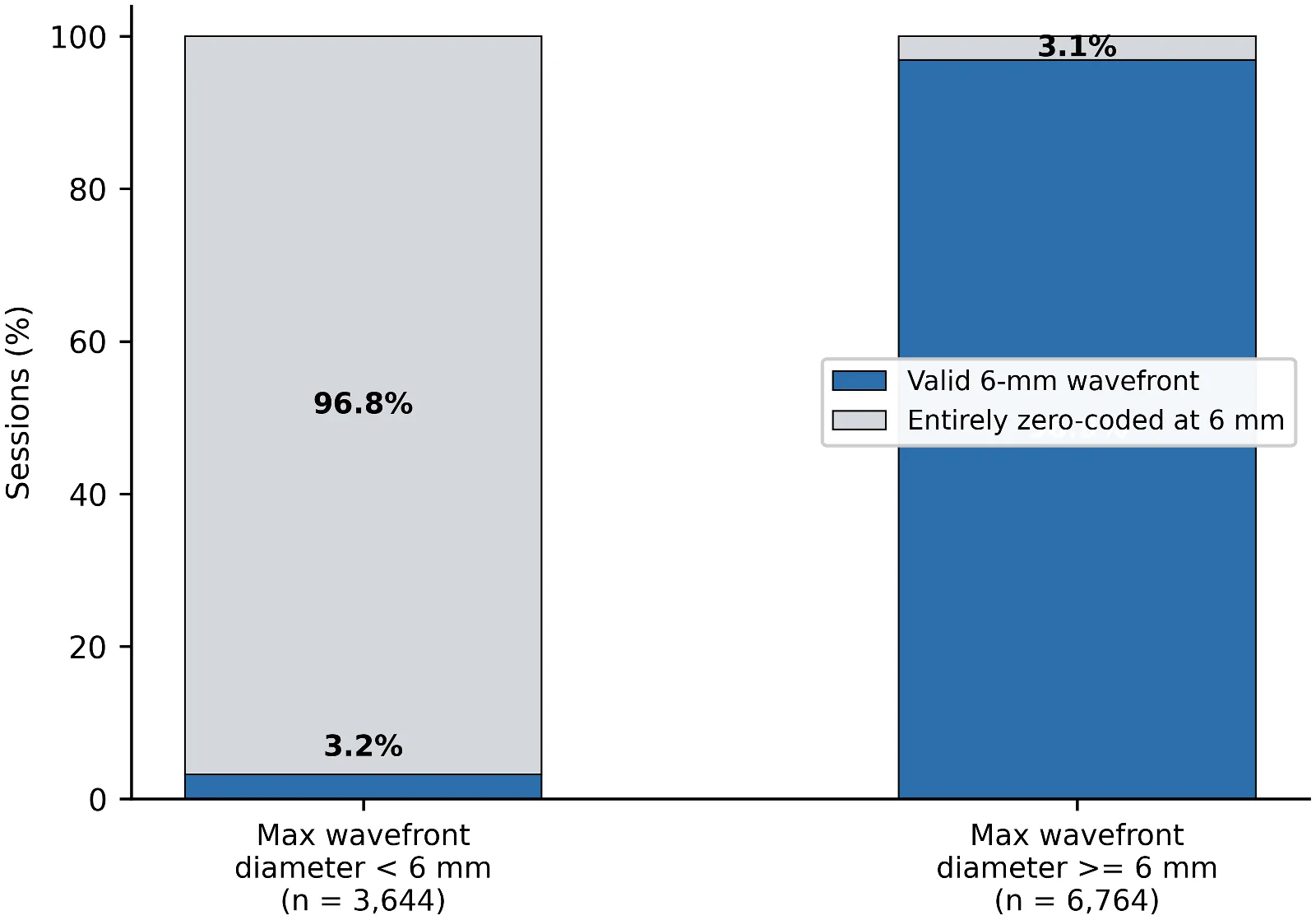
**Pupil-support gate.** Proportion of valid versus entirely zero-coded 6-mm wavefronts by maximum wavefront diameter. A diameter below 6 mm yields a valid 6-mm export in only 3.2% of sessions.

### Quality-Control Sensitivity

Skipping the registry scan-selection workflow and retaining every numeric raw scan markedly worsened repeatability (Table 5). The 4-mm HOA repeatability coefficient increased from 0.033 to 0.083 µm, and the 6-mm HOA repeatability coefficient increased from 0.050 to 0.257 µm, a roughly 5-fold degradation at 6 mm. The scan-selection algorithm and zero-code exclusion are therefore part of the validated measurement workflow, not optional post-processing, and the reported repeatability is conditional on that workflow.

**Table 5.** Quality-control Sensitivity: Repeatability Coefficient With and Without Scan Selection

Metric	Aperture	RC, QC-Selected	RC, All Numeric Scans
HOA RMS	4 mm	0.033 µm	0.083 µm
HOA RMS	6 mm	0.050 µm	0.257 µm

### Cross-Domain and Clinical Agreement

OSIRIS whole-eye and MS-39 total-corneal wavefront were correlated but not interchangeable (Table 6; Figs. 3, 4,^5^). For 6-mm HOA RMS in pupil-supported eyes, the MS-39-minus-OSIRIS bias was +0.066 µm with 95% LoA of −0.279 to +0.410 µm, Pearson *r* = 0.670, and CCC = 0.623. For 6-mm spherical aberration, the bias was −0.185 µm with 95% LoA of −0.434 to +0.065 µm, Pearson *r* = 0.710, and CCC = 0.442. These are cross-domain differences between whole-eye and total-corneal optics, not evidence that either device is inaccurate. As expected from Zernike aperture scaling, OSIRIS 6-mm HOA RMS exceeded 4-mm HOA RMS by a mean of +0.256 µm.

**Figure 3.**
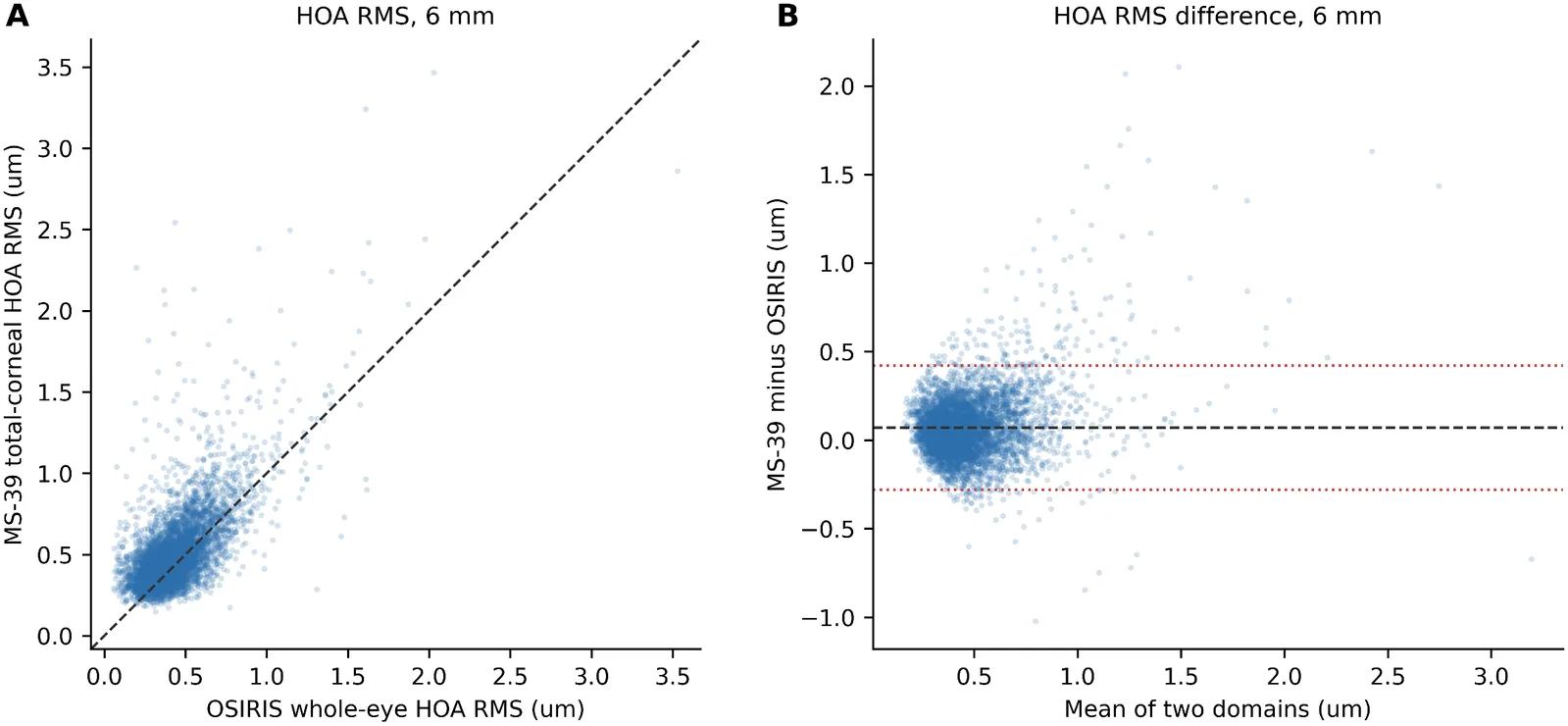
**OSIRIS whole-eye versus MS-39 total-corneal 6-mm HOA RMS.** (**A**) Scatter with identity line. (**B**) Bland-Altman plot with mean bias and 95% limits of agreement.

**Figure 4.**
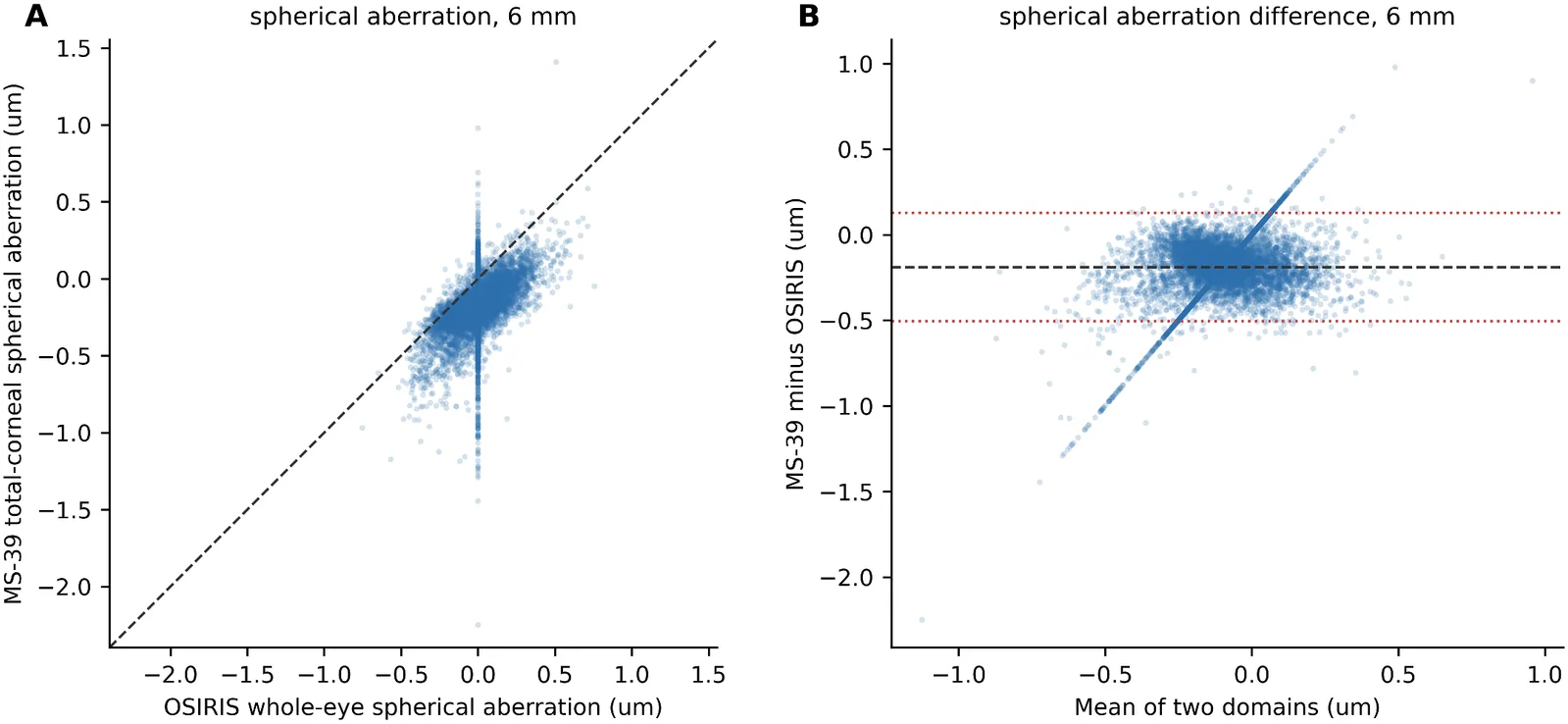
**OSIRIS whole-eye versus MS-39 total-corneal 6-mm spherical aberration.** (**A**) Scatter with identity line. (**B**) Bland-Altman plot.

**Figure 5.**
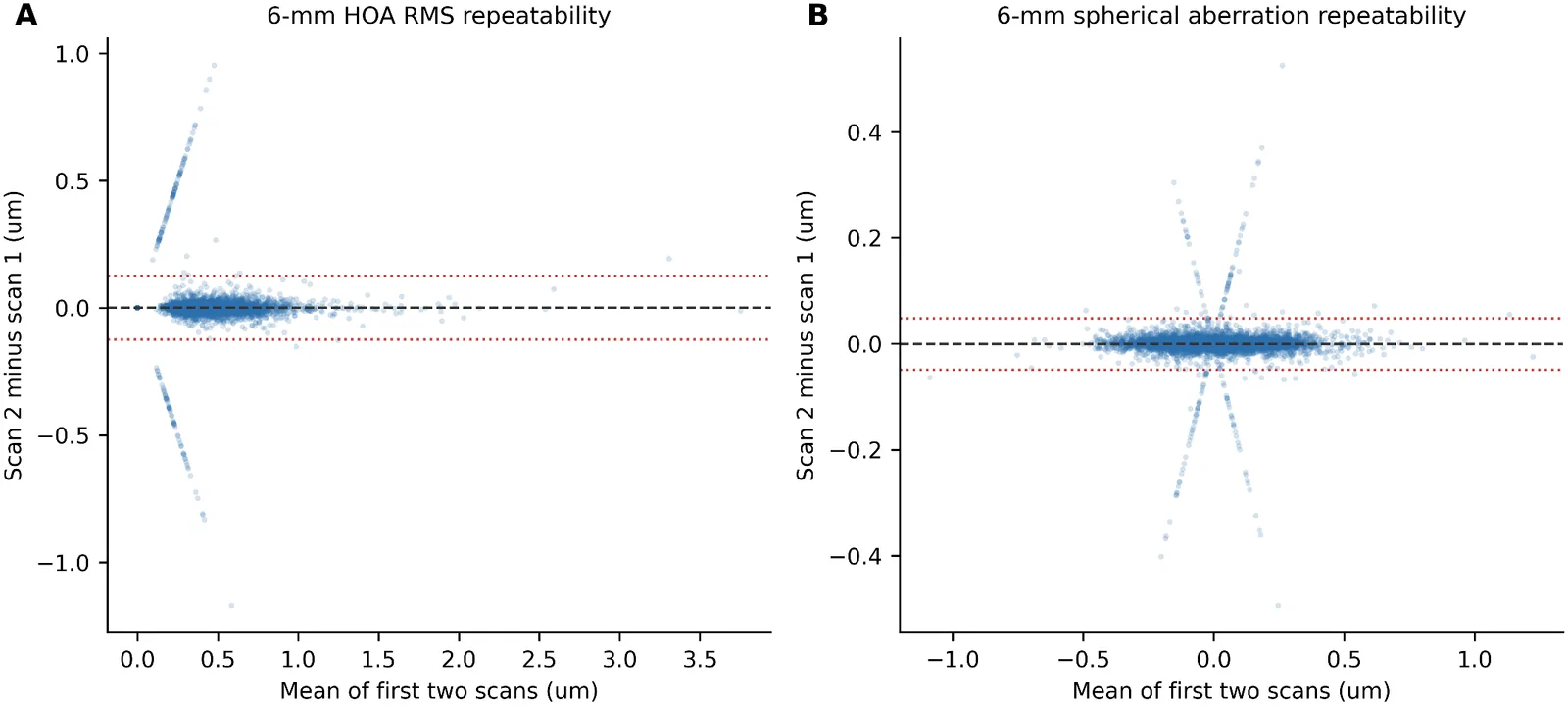
**Within-session repeatability Bland-Altman plots.** (**A**) 6-mm HOA RMS. (**B**) 6-mm spherical aberration.

**Figure 6.**
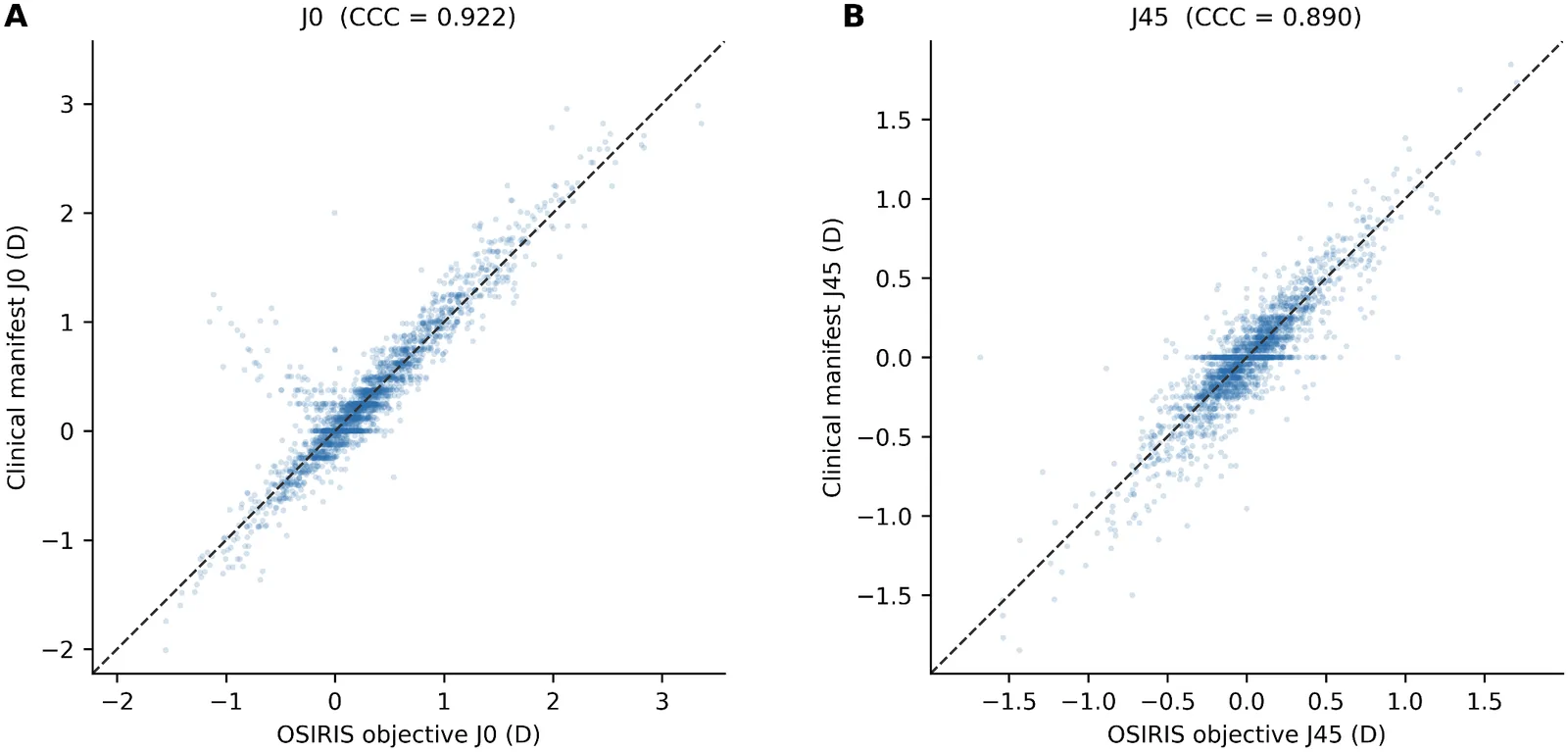
**Preoperative astigmatic power-vector agreement, 4 mm.** Clinical manifest versus OSIRIS objective Thibos power vectors in preoperative eyes with a recorded clinical axis (*n* = 2,373), with identity line and Lin concordance correlation coefficient (CCC). (**A**) J0, the with-the-rule/against-the-rule (0/90 degree) astigmatic component, CCC = 0.922. (**B**) J45, the oblique (45/135 degree) astigmatic component, CCC = 0.890.

The comparison of clinical manifest and OSIRIS objective refraction was descriptive, because a substantial fraction of clinical values matched the expected rounding of OSIRIS objective values (for example, sphere and cylinder matched expected rounding in 52% and 74% of 4-mm cases), so the clinical reference may not be fully independent of the objective measurement. With that caveat, agreement was high (Table 7). At 4 mm, sphere bias was −0.109 D (95% LoA = −1.054 to +0.836 D and CCC = 0.965) and cylinder bias was −0.001 D (95% LoA = −0.402 to +0.399 D and CCC = 0.956); spherical-equivalent bias was −0.101 D (95% LoA = −1.089 to +0.886 D and CCC = 0.965). At 6 mm, agreement was slightly lower, as expected from the smaller pupil-supported subset and larger aperture (sphere CCC = 0.944 and cylinder CCC = 0.878).

Astigmatism agreement was assessed on power vectors in the preoperative subset with a recorded clinical axis (2373 eyes at 4 mm and 1715 at 6 mm; Table 8). The astigmatic vectors agreed well (Fig. 6): at 4 mm, J0 bias was +0.014 D (CCC = 0.922) and J45 bias was −0.009 D (CCC = 0.890); at 6 mm, J0 CCC was 0.869 and J45 CCC was 0.836. The spherical-equivalent vector M carried a preoperative bias of approximately −0.36 to −0.38 D, consistent with manifest refraction being more myopic than the objective wavefront in unoperated eyes. Raw axis agreement was dominated by low-cylinder eyes, in which axis is poorly defined (Fig. 7). Across all eyes the median absolute axis difference was 5.6 degrees at 4 mm, but restricting to eyes with at least 0.50 D of cylinder on both methods reduced the mean absolute difference from 13.4 to 7.9 degrees and raised within 10-degree agreement from 67.9% to 83.1%; at a 1.00 D threshold, the median absolute axis difference was 3.1 degrees with 91.1% of eyes agreeing within 10 degrees (see Table 8). The same pattern held at 6 mm.

**Figure 7.**
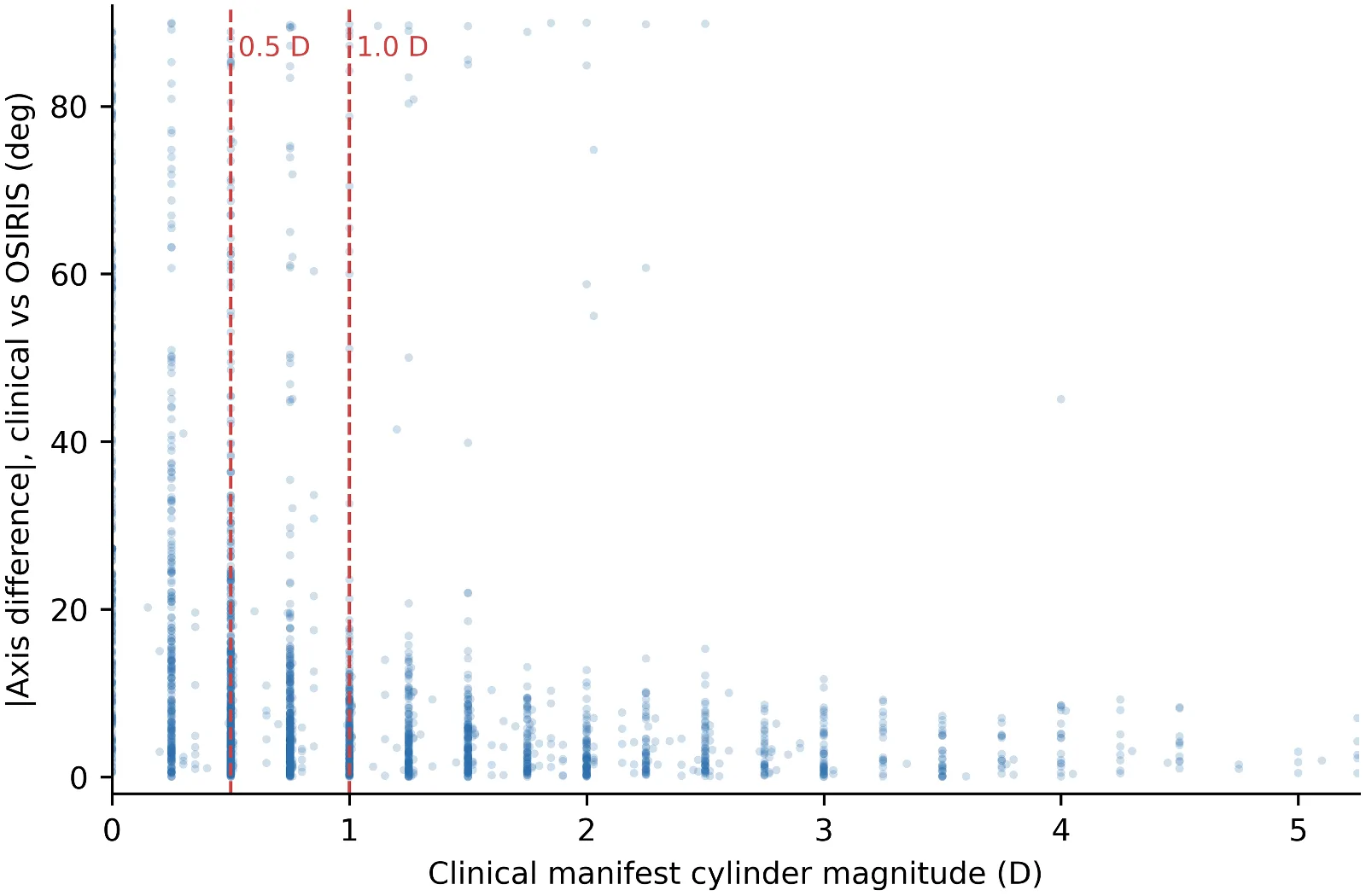
**Axis agreement versus cylinder magnitude, 4 mm.** Absolute clinical-versus-OSIRIS axis difference against clinical cylinder magnitude. Agreement improves sharply as cylinder increases; dashed lines mark the 0.50 and 1.00 D thresholds. Axis is mathematically ill-defined near zero cylinder.

## Discussion

Across thousands of sessions in a live clinical registry, OSIRIS demonstrated excellent within-session repeatability of whole-eye wavefront measurement. The repeatability coefficient, the interval within which 95% of differences between 2 same-session measurements of the same eye are expected to fall, was 0.033 µm for 4-mm HOA RMS and 0.050 µm for pupil-supported 6-mm HOA RMS. Both values sit at or below the 0.10-µm figure commonly cited as a benchmark for an aberrometrically relevant HOA change,^1^ and at the tight end of the values reported for any aberrometer (Table 1). Framed for the surgeon, the RC bounds what two same-session acquisitions can be expected to disagree by, so a same-session difference exceeding roughly 0.03 to 0.05 µm, depending on aperture and stratum, is unlikely to reflect measurement variability alone. This is a necessary precision condition, not a sufficient criterion for action: a longitudinal change adds day-to-day biological and alignment variation that within-session repeatability does not capture, and the 0.10-µm benchmark is not an established boundary of clinical significance. These coefficients characterize the OSIRIS acquisition and QC workflow as operated here, not an intrinsic property of the pyramidal sensor in isolation.

**Table 6.** Cross-Domain and Clinical Agreement

Comparison	*N*	Bias	95% LoA	Pearson r	CCC
MS-39 total-corneal minus OSIRIS whole-eye HOA RMS, 6 mm (pupil-supported)	5,644	+0.066 µm	−0.279 to +0.410 µm	0.670	0.623
MS-39 total-corneal minus OSIRIS whole-eye spherical aberration, 6 mm	5,798	−0.185 µm	−0.434 to +0.065 µm	0.710	0.442
OSIRIS 6-mm minus 4-mm HOA RMS (aperture effect)	6,167	+0.256 µm	−0.029 to +0.540 µm	0.710	0.248

Bias is method_x minus method_y. Cross-domain rows compare different optical domains and assess concordance, not interchangeability.

**Table 7.** Agreement of Refraction: Clinical Manifest Minus OSIRIS Objective (Descriptive)

Metric	Aperture	n	Bias	95% LoA	Pearson r	CCC
Sphere	4 mm	8,239	−0.109 D	−1.054 to +0.836 D	0.969	0.965
Cylinder	4 mm	8,238	−0.001 D	−0.402 to +0.399 D	0.957	0.956
Spherical equivalent	4 mm	8,229	−0.101 D	−1.089 to +0.886 D	0.969	0.965
Sphere	6 mm	5,378	−0.187 D	−1.412 to +1.037 D	0.950	0.944
Cylinder	6 mm	5,377	−0.042 D	−0.703 to +0.619 D	0.882	0.878
Spherical equivalent	6 mm	5,370	−0.204 D	−1.490 to +1.083 D	0.949	0.943

Clinical manifest refraction may not be fully independent of the OSIRIS objective value (rounding overlap), so this is a consistency check, not an accuracy validation. Full visit-stratified results (PREOP, POM1, POM3, POM6) are in the analysis package.

**Table 8.** Preoperative Astigmatism and Axis Agreement: Clinical Manifest Versus OSIRIS Objective

Component	Aperture	*n*	Bias	95% LoA	Pearson r	CCC
J0	4 mm	2,373	+0.014 D	−0.441 to +0.469 D	0.923	0.922
J45	4 mm	2,373	−0.009 D	−0.298 to +0.281 D	0.892	0.890
M (SEQ)	4 mm	2,373	−0.358 D	−1.720 to +1.004 D	0.932	0.912
J0	6 mm	1,715	+0.018 D	−0.552 to +0.588 D	0.870	0.869
J45	6 mm	1,715	−0.022 D	−0.359 to +0.315 D	0.840	0.836
M (SEQ)	6 mm	1,715	−0.383 D	−2.251 to +1.486 D	0.867	0.848
Axis Agreement (Acute Angular Difference) by Cylinder Magnitude, Both Methods Above Threshold						
Aperture	Cylinder Threshold	*n*	Mean |ΔAxis|	Median |ΔAxis|	Within 10 Deg	Within 15 Deg

4 mm	None	2,373	13.4 deg	5.6 deg	67.9%	77.5%
4 mm	≥0.50 D	1,571	7.9 deg	4.0 deg	83.1%	91.9%
4 mm	≥1.00 D	865	6.0 deg	3.1 deg	91.1%	96.4%
6 mm	None	1,715	15.0 deg	6.7 deg	63.0%	73.4%
6 mm	≥0.50 D	1,094	9.2 deg	4.5 deg	79.2%	88.7%
6 mm	≥1.00 D	583	6.9 deg	3.3 deg	89.0%	95.2%

Power vectors (clinical minus OSIRIS), preoperative eyes with a recorded clinical axis. Clinical manifest axis was charted only at the preoperative visit; the analysis is therefore preoperative. CIs are patient-level cluster bootstrap.

These results are consistent with, and generally tighter than, prior pyramidal-sensor data. Bench validation of the pyramidal sensor reported root-mean-square measurement errors of approximately 0.01 to 0.02 µm for spherical aberration and coma on a model eye.^2^ The first clinical repeatability study, in 53 healthy eyes, reported ICCs of at least 0.798 at 4 mm and at least 0.850 at 6 mm.^3^ A larger study in 76 healthy and 72 keratoconic eyes reported ICCs above 0.9 for total lower- and higher-order aberrations in both groups, with within-subject SDs in keratoconus of approximately 0.062 µm for HOA RMS and 0.022 µm for spherical aberration.^4^ The present registry extends this evidence by roughly two orders of magnitude in sample size and reports even tighter repeatability coefficients, which reflects both the QC scan-selection workflow and the stability conferred by large numbers. The real-world cohort, spanning untreated myopic eyes and the same eyes across multiple postoperative time points, further supports the external validity of the estimate within the myopic treatment pathway.

The stratified analysis adds a caution to any pooled figure. Preoperative eyes were the noisier stratum for the HOA metrics, with postoperative-to-preoperative SD ratios of 0.60 to 0.74, the opposite direction from a tear-film-driven expectation for operated eyes. A plausible contributor is first-visit measurement unfamiliarity, because preoperative acquisitions are typically a patient's first encounter with the instrument, whereas postoperative visits repeat a practiced procedure; the design cannot isolate the mechanism. Whatever its source, the heterogeneity means the pooled coefficients bound neither stratum well, and Table 3 values should be preferred when a specific clinical context is at stake.

Placed against Hartmann-Shack, ray-tracing, and slit-skiascopy devices, OSIRIS repeatability is comparable to or better than published values, with the important caveat that no same-eye multi-device arm was run here, so these are cross-study comparisons rather than head-to-head measurements. In 92 healthy eyes at a 4-mm aperture, a ray-tracing aberrometer (iTrace) achieved whole-eye HOA repeatability limits of 0.105 µm or less, and a Hartmann-Shack aberrometer (Topcon KR-1 W) achieved 0.079 µm or less.^9^ A three-device comparison found that whole-eye HOA RMS repeatability was similar across the Pentacam AXL Wave, iTrace, and OPD-Scan III, with the Pentacam AXL Wave and OPD-Scan III outperforming the iTrace for total-RMS repeatability.^10^ Spherical aberration is consistently the most repeatable HOA term across technologies, a pattern the present data reproduce. Older and lower-sampling instruments have reported substantially larger spherical-aberration variability, illustrating that repeatability is device- and generation-dependent and cannot be assumed to transfer from one aberrometer to another.^1^^,^^9^

The pupil-support and zero-code gate is the study's principal methodological contribution beyond OSIRIS itself. The finding that a maximum wavefront diameter below 6 mm produced a zero-coded 6-mm export in 96.8% of sessions, whereas a diameter at or above 6 mm produced a valid export in 96.9%, demonstrates that large-pupil aberrometry silently fails when the pupil does not support the requested aperture. Because the stratified analysis showed no material degradation of HOA repeatability below 6 mm once zero-coded exports were removed, the risk is not subtle imprecision but the propagation of null exports as if they were true low-aberration measurements. The gate, requiring both a valid non-zero wavefront and a maximum wavefront diameter of at least 6.0 mm, is a simple and transferable safeguard that applies to any large-pupil wavefront analysis, not only to OSIRIS. Its claim is deliberately limited: it certifies that a 6-mm export exists and is valid, not that the peripheral wavefront is true, which would require an independent optical reference. Consistent with an availability rather than a precision mechanism, repeatability showed no deterioration just above the threshold; the 6.00 to 6.24 mm band was the tightest of all diameter bands.

A related methodological point is that the reported repeatability is conditional on the scan-selection workflow. Retaining every numeric raw scan degraded the 6-mm HOA repeatability coefficient roughly 5-fold. Repeatability figures for any aberrometer should therefore be interpreted together with the acquisition and selection procedure that produced them, and that procedure should be reported so that others can reproduce it. The precision reported here is a property of the acquisition-plus-selection system as a whole, and portability to sites that do not operate an equivalent workflow should not be assumed. This is consistent with the general observation that aberrometer precision is sensitive to tear-film state, fixation, and acquisition discipline.^9^

Objective refraction from OSIRIS agreed closely with clinical manifest refraction. Sphere, cylinder, and spherical-equivalent concordance were high (CCC = 0.88 to 0.97), and preoperative astigmatism agreed well when analyzed as power vectors (J0 and J45 CCC = 0.84 to 0.92), avoiding the circular-scale artifacts that distort raw axis comparisons. Cylinder axis agreement was strongly dependent on cylinder magnitude, as expected: axis is mathematically ill-defined near zero cylinder, so the apparent axis scatter in low-astigmatism eyes reflects the definition of the metric rather than device disagreement. Once eyes with at least 1.00 D of cylinder were considered, the median axis difference fell to about 3 degrees with more than 90% of eyes within 10 degrees. This has a direct clinical reading: OSIRIS objective astigmatism is trustworthy for planning in eyes with clinically meaningful cylinder, and axis values in near-spherical eyes should be treated as indeterminate rather than as measurement error. Because clinical manifest axis was documented only preoperatively in this registry, and because manifest refraction may not be fully independent of the objective value, this comparison is a preoperative consistency check rather than an accuracy validation.

The MASI, a device-derived composite shown to be associated with contrast sensitivity after keratorefractive surgery,^5^ was reported with excellent within-session repeatability here (ICC = 0.976 and RC = 7.392). This gives the metric the precision foundation required for the longitudinal quality-of-vision monitoring proposed for it. The paper introducing MASI identified reproducibility as an open question^5^; the present data establish its within-session, within-operator precision, whereas inter-device, inter-operator, and multicenter reproducibility remain outstanding.

Finally, OSIRIS whole-eye wavefront and MS-39 total-corneal wavefront were correlated but not interchangeable. This is expected: the two instruments measure different optical domains, the whole eye versus the total cornea, so systematic, aperture-dependent differences are the correct result rather than a defect of either device. The same conclusion, that devices based on different measurement principles or optical domains should not be used interchangeably, has been reached repeatedly in the aberrometer-comparison literature.^9^^,^^10^ Clinically, the result answers a planning question rather than ranking instruments: whole-eye wavefront is the appropriate input for wavefront-guided strategies and corneal wavefront for corneal- and topography-guided strategies, the two should not be mixed within a single longitudinal series, and one cannot substitute for the other in treatment planning.

This study has limitations. It is retrospective and single-center, and used a single device unit, so between-unit variation was not assessed. It reports within-session repeatability only; inter-session, inter-day, and inter-operator reproducibility were not evaluated, and aberrations are known to fluctuate over time, so reproducibility is expected to be lower than repeatability.^9^ Repeated scans within a session were acquired by the same operator, although four operators contributed across the registry, so the estimates reflect routine multi-operator practice rather than a single examiner. Repeatability is not accuracy; no independent optical gold standard was available to establish trueness in vivo. The cohort comprised myopic and myopic astigmatic eyes studied before and after lenticule extraction, so it was not a homogeneous single-timepoint sample, although preoperative and postoperative repeatability are reported separately (see Table 3), and the findings should not be extrapolated to hyperopic eyes, eyes with previous corneal refractive surgery, or pseudophakic eyes, in which pupil size, tear film, higher-order aberration magnitude, and internal optics differ substantially; those populations are the subject of planned separate reports. The clinical-refraction reference was not fully independent of the objective measurement, and clinical manifest axis was available only preoperatively, so the astigmatism and axis agreement analyses are preoperative. MASI is a device-specific composite reported on a registry-scaled basis and should be interpreted accordingly.

## Conclusions

OSIRIS provides excellent within-session repeatability for 4-mm and pupil-supported 6-mm whole-eye wavefront measurement under the described acquisition and quality-control workflow, with repeatability coefficients at or below the commonly cited 0.10-µm benchmark for higher-order aberrations. Six-millimeter analysis requires explicit pupil-diameter and zero-code gating, which is a transferable safeguard for large-pupil aberrometry generally. OSIRIS whole-eye wavefront and MS-39 total-corneal wavefront should not be treated as interchangeable.

## Supplementary Material

Supplement 1
