# Supplementary material for: Repeatability and Cross-Domain Agreement of Pyramidal-Sensor Ocular Wavefront Measurements in Myopic Eyes: A Registry Validation of OSIRIS
Source: Transl Vis Sci Technol. 2026 Oct 29;15(10):49. doi: 10.1167/tvst.15.10.49 (PMC13726724; doi:10.1167/tvst.15.10.49)
Supplement: Supplementary file 1 [file tvst-15-10-49_s001.docx]

# Supplementary Material

**Repeatability and cross-domain agreement of pyramidal-sensor ocular wavefront measurements in myopic eyes: a registry validation of OSIRIS**

Gabrić I, Modrić IS, Grulović F

## Supplement S1. Acquisition protocol

**Cohort scope.** The study population comprised myopic and myopic astigmatic eyes undergoing keratorefractive lenticule extraction (preoperative manifest spherical equivalent -12.75 to -0.60 D). Hyperopic eyes, eyes with previous corneal refractive surgery, and pseudophakic eyes were excluded and are the subject of planned separate reports.

All OSIRIS acquisitions followed a single standardized registry workflow.

**Room conditions.** Measurements were performed in a darkened examination room with no additional illumination beyond that of the device itself. No pharmacologic dilation was used; pupil size was the patient’s natural scotopic diameter.

**Sequence within the visit.** Aberrometry was always performed first in the examination sequence, before topography, tomography, and pupillometry. This ordering was deliberate: it ensures that wavefront acquisition occurs before the eye is exposed to the illumination of subsequent instruments, so that pupil diameter and tear-film state at the time of wavefront capture are not influenced by preceding tests.

**Scan protocol.** Each eye was scanned a minimum of three times. If three acquisitions of acceptable quality were not obtained, the sequence was repeated until three good scans were available. The registry scan-selection workflow then retained the representative scans used for clinical averaging; these retained scans form the primary repeatability cohort described in the main text.

**Operators.** All acquisitions across the registry were performed by four highly trained optometrists. Within any single session, the repeated scans of a given eye were acquired by the same operator, so the repeatability reported in the main text is within-operator, within-session. Because four operators contributed across the registry as a whole, the estimate reflects routine multi-operator clinical practice rather than the performance of a single examiner.

**Devices.** OSIRIS pyramidal wavefront-sensor aberrometer and MS-39 anterior-segment optical coherence tomographer with integrated Placido topography (both CSO, Florence, Italy), operated under Phoenix software version 4.1.4.7. All captures analyzed in this study were acquired under this software version.

## Supplement S2. Aperture definition and pupil sampling

**Aperture definition.** Analysis apertures are defined relative to the maximum wavefront capture diameter of each acquisition. The device automatically samples the largest pupil available at the time of capture, up to a ceiling of 9.0 mm. The maximum wavefront diameter recorded for each scan (max_wavefront_diameter_mm) is therefore an acquisition property, not a fixed setting.

**Typical pupil sizes in this cohort.** The registry population is predominantly young refractive-surgery candidates, whose natural scotopic pupil diameters typically fall between 6 and 7 mm. Consequently the majority of sessions support a 6-mm analysis aperture. A minority of eyes do not achieve a sufficiently large pupil under these conditions, and in those eyes a 6-mm wavefront cannot be reconstructed.

**Consequence for 6-mm analysis.** This is the direct physical basis for the pupil-support and zero-code gate described in the main text. When the maximum wavefront diameter is below 6.0 mm, the device does not return a valid 6-mm wavefront and instead exports a zero-coded result. Such exports are not true low-aberration measurements and were excluded rather than treated as zeros. The 6-mm analysis gate therefore required both a valid non-zero wavefront and a maximum wavefront diameter of at least 6.0 mm. The 4-mm gate required a valid non-zero wavefront only, since 4 mm is supported in essentially all acquisitions.

## Supplement S3. Metric dictionary

Fields as they appear in the released anonymized scan-level dataset (selected_repeat_scan_data_anonymized.csv). Zernike coefficients follow the OSA ordering and normalization standard.

| Field | Definition |
| --- | --- |
| patient_id | Anonymized patient token |
| eye_id | Anonymized eye token |
| laterality | OD or OS |
| visit_label | PREOP, POM1, POM3, POM6, or POM12 |
| days_from_surgery | Integer days relative to surgery (no calendar dates) |
| session_id | Within-session cluster identifier (patient x laterality x date x time) |
| scan_order_source | Provenance of scan ordering within the session |
| max_wavefront_diameter_mm | Maximum wavefront capture diameter for the scan |
| pupil_supports_4mm_flag, pupil_supports_6mm_flag | Whether maximum diameter supports the stated aperture |
| hoa_rms_4mm_um, hoa_rms_6mm_um | Higher-order aberration RMS at the stated aperture (um) |
| coma_rms_4mm_um, coma_rms_6mm_um | Coma RMS at the stated aperture (um) |
| trefoil_rms_4mm_um, trefoil_rms_6mm_um | Trefoil RMS at the stated aperture (um) |
| spherical_aberration_4mm_um, spherical_aberration_6mm_um | Spherical aberration, Zernike Z(4,0), OSA index j = 12 (um); native sign retained |
| z3m1_4mm_um, z3p1_4mm_um, z3m1_6mm_um, z3p1_6mm_um | Vertical and horizontal coma coefficients Z(3,-1) and Z(3,1) (um); native sign retained |
| objective_sphere_*_D, objective_cylinder_*_D, objective_axis_*_deg, objective_seq_*_D | Objective refraction at the stated aperture (D, D, degrees, D) |
| valid_4mm_flag, valid_6mm_flag | Whether a valid non-zero wavefront was returned at the stated aperture |
| zero_coded_4mm_flag, zero_coded_6mm_flag | Whether the export was entirely zero-coded at the stated aperture |
| candidate_scan_count, good_candidate_scan_count | Raw and quality-passing scan counts contributing to the session |
| plausibility_outliers_dropped | Count of scans removed by the robust plausibility rule (>3.5 MAD, MASI) |

**Derived metrics reported in the main text but not stored per scan.** The Strehl ratio and the Micro Aberration Scatter Index (MASI) are device-reported composites. MASI is computed over the central 3 mm zone and is reported multiplied by 1000 following the registry convention (Gabric et al., BMC Ophthalmol 2026, doi:10.1186/s12886-026-05118-8). Astigmatism agreement used Thibos power vectors, computed as J0 = -(C/2)cos(2a), J45 = -(C/2)sin(2a), and M = S + C/2, where S is sphere, C is cylinder, and a is axis in radians.
